# A systematic review of the methodological quality of randomised trials in IBD surgery

**DOI:** 10.1111/codi.70531

**Published:** 2026-06-18

**Authors:** Kelsey Aimar, Aditya Gaur, Michael Peirson, Josephine Walshaw, Thomas D. Pinkney, Matthew J. Lee

**Affiliations:** ^1^ Belfast Health and Social Care Trust Belfast UK; ^2^ Somerset NHS Foundation Trust Taunton UK; ^3^ Bradford Teaching Hospitals NHS Foundation Trust Bradford UK; ^4^ Leeds Institute of Medical Research University of Leeds Leeds UK; ^5^ University Hospitals Birmingham NHS Foundation Trust Birmingham UK; ^6^ Department of Applied Health Sciences, College of Medicine & Health University of Birmingham Birmingham UK

**Keywords:** Crohn's disease, inflammatory bowel disease, methodology, randomised trial, surgery, systematic review, ulcerative colitis

## Abstract

**Aims:**

This systematic review aimed to evaluate the design, statistical robustness and real‐world applicability of randomised controlled trials in IBD surgery.

**Methods:**

A systematic review of RCTs involving adult patients with IBD undergoing surgical interventions was conducted. Databases and registries were searched from 2005 to 2025. Dual screening and extraction were performed. Trials were appraised using RoB‐2, PRECIS‐2 and fragility index. The study was registered with PROSPERO (CRD420251058758).

**Results:**

From 5803 abstracts, 18 published RCTs and 26 registered but unpublished trials were identified. Published RCTs had a median planned sample size of 102 participants (IQR 80–143) and recruited a median of 89 (IQR 55–114), with frequent under‐recruitment (7/18, 39%) and early termination (5/18, 28%). Methodological appraisal revealed that most studies (12/18, 67%) had some concern for bias on RoB‐2 assessment. PRECIS‐2 evaluation showed a tendency towards pragmatic designs. The median fragility index was 3 (IQR 1–6). Of registered, unpublished trials, median planned sample size was 155 (IQR 93–196), with the largest study having a planned enrolment of 550 participants.

**Conclusions:**

Surgical RCTs in IBD face challenges across design and delivery, such as modest sample sizes, under‐recruitment and statistical fragility. The findings from this review highlight the importance of larger, multicentre and collaborative trials to strengthen the evidence base in IBD surgery.

**What this paper adds to the literature?**

This paper has synthesised published and planned surgical trials in IBD. It has highlighted some of the vulnerabilities of trial design in this field and suggests strategies to address these.

## INTRODUCTION

Inflammatory bowel disease (IBD), encompassing Crohn's disease (CD) and ulcerative colitis (UC), is an unpredictable, relapsing–remitting, chronic inflammatory condition of the gastrointestinal tract. Medical therapy with immunosuppressants and biologics forms the mainstay of IBD management, yet surgery remains an essential component of treatment for many patients. An estimated 26%–47% of CD patients and 6%–10% of UC patients require abdominal surgery 10 years from their diagnosis [1, 2, 3, 4]. Common indications for surgery in IBD include medically refractory disease and complications including intestinal strictures, bowel perforation, abscesses, fistulas or malignancy [5]. The varied presentation of IBD and its complications necessitates a range of operative strategies that are equally diverse. Amidst this complexity, surgical management of IBD must remain grounded in evidence‐based principles. For colorectal surgeons managing IBD, surgical RCTs are often the highest level of evidence available, yet their reliability and generalisability remain uncertain. Consequently, there remain questions about optimal anastomotic technique, mesenteric resection [6] and optimum perianal interventions [7].

As the gold standard in clinical research, randomised controlled trials (RCTs) provide the highest level of evidence to evaluate treatment effects and underpin modern evidence‐based practice [8] While numerous RCTs provide evidence for the medical management of IBD, particularly with biologic agents [9, 10], surgical RCTs remain relatively scarce [11, 12]. Given the pivotal role of RCTs in shaping treatment guidelines, a focused appraisal of the methodological rigour and clinical applicability of contemporary surgical trials in IBD is warranted. Robustness and generalisability can be considered in different ways. Most recently, the fragility index has been utilised to assess how resilient a significant effect is when event rates are changed [13]. When statistical significance is impacted with a small change in event rates, this may mean the finding is not robust. For assessment of external validity, the PRagmatic Explanatory Continuum Indicator Summary (PRECIS‐2) [14] tool has been proposed as a tool to support these assessments.

This systematic review aimed to evaluate the design, statistical robustness and real‐world applicability of randomised controlled trials in IBD surgery. Insights from this analysis may help inform the design of future high‐quality trials.

## METHODS

A systematic review was performed. This study was registered on PROSPERO (CRD420251058758), conducted with reference to the Cochrane Handbook for Systematic Reviews of Interventions [15], and is reported in accordance with the PRISMA guidelines [16].

### Study eligibility

Randomised controlled trials involving adult patients (≥18 years) with IBD were included if they featured one or more surgical treatment arms. Eligible interventions involved surgical procedures for ulcerative colitis or intestinal or perianal Crohn's disease, including, for example ileal pouch‐anal anastomosis, proctectomy, subtotal colectomy, ileocolic resection, small bowel resection, appendicectomy and strictureplasty. Endoscopic intervention alone was not considered a surgical intervention. Any comparator was permitted provided at least one treatment arm involved surgical intervention. Only studies published in English from 2005 onward were considered. Studies were excluded if they lacked a surgical treatment arm, used a non‐randomised design or involved mixed patient populations in which data for IBD patients could not be separately extracted.

### Search strategy

Systematic searches were developed with the support of the Evidence Support team at the Royal College of Surgeons of England. These searched published literature in Cochrane CENTRAL, Embase and MEDLINE databases for trials published between 1 January 2005 and 21 May 2025. Clinical trial registries (ClinicalTrials.gov and ISRCTN) were systematically searched using disease keywords and surgery to capture additional eligible but unpublished studies. The sample search strategy is available in the Supplementary Information.

### Study selection and data extraction

All search results were imported into Covidence systematic review software (Veritas Health Innovation, Australia), and duplicates were removed. Records were independently screened in duplicate by two of four reviewers (KA, AG, JW, MP), and any disagreements were resolved by a fifth reviewer (MJL). Additionally, registered clinical trials meeting inclusion criteria were hand‐searched to identify any publications not captured in the initial database search. Full texts of studies deemed potentially eligible were retrieved and assessed using the same dual‐review process.

Data extraction focused on five main areas: study characteristics (title, author, year, country), trial design (setting, participants, interventions, loss to follow‐up, intended sample size, study power, intended effect size), primary outcome data for calculation of the fragility index (number of participants with and without the primary endpoint in each treatment arm), PRECIS‐2 domain scores and risk of bias. Using a predefined electronic data extraction form, four reviewers (KA, AG, JW, MP) independently extracted data in pairs. Discrepancies were resolved by consensus or by a fifth review (MJL) in cases of disagreement. In our study, loss to follow‐up comprised all randomised participants who were not analysed for the primary outcome for any reason. The primary outcome measure was defined as that which was explicitly stated as the primary outcome in the article. If not specified, then the outcome used for the sample size calculation was selected. If neither applied, then the first outcome reported in the results section was used.

### Calculation of the fragility index

For binary outcomes, fragility and reverse fragility were calculated using the Fragility package [17] in R statistics [18]. This quantifies how many event changes are required to alter the statistical significance of a result, expressed as the fragility index. Larger indices suggest greater robustness, with a median value of 8 reported across RCTs in the literature [13]. Although methods have been proposed to assess fragility in continuous outcomes [19], this is not widely undertaken.

### Appraisal of trial pragmatism

The PRECIS‐2 tool was used to evaluate each study by rating key trial design domains on a 5‐point Likert scale ranging from (1) very explanatory to (5) very pragmatic [14]. The nine PRECIS‐2 domains comprise: eligibility, recruitment, setting, organisation, flexibility (delivery), flexibility (adherence), follow‐up, primary outcome and primary analysis. For this review, we modified the tool by excluding the ‘flexibility (adherence)’ domain, which pertains to the extent of participant adherence to the assigned intervention. In the context of surgical RCTs, the intervention is delivered intra‐operatively leaving no opportunity for variation in participant active engagement, thereby rendering this domain inapplicable. Ratings from 1 to 5 were assigned to each of the remaining eight domains. This was assessed by two reviewers, and adjudication of conflicts was undertaken by MJL.

### Assessment of risk of bias in included studies

The Risk of Bias (RoB 2) tool [20] was used to assess the included studies for the presence of bias. Studies underwent dual assessment with conflict resolution by a third reviewer.

## RESULTS

### Study selection

**FIGURE 1:**
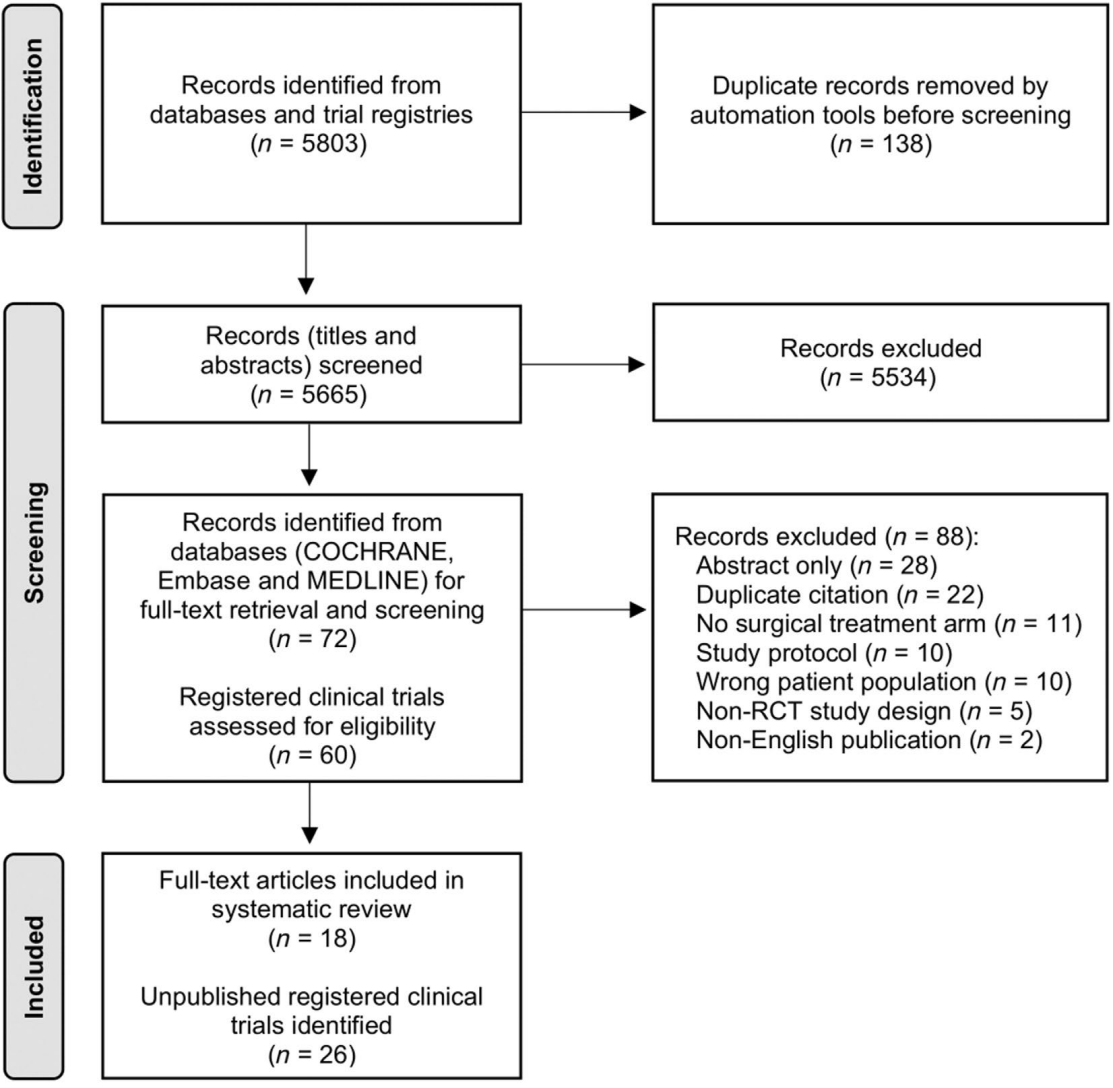
PRISMA flow diagram.

5803 records were identified from database and trial registry searches (Figure 1). After automated removal of duplicates by Covidence software, 5665 titles and abstracts were screened. Of these, 132 records were included for full‐text assessment of eligibility—72 from databases and 60 from clinical trial registries. Ultimately, 18 published RCTs were included in the systematic review, and 26 additional registered but unpublished trials were identified.

### Characteristics of included trials

Table 1 presents summary characteristics of the 18 included studies. The majority (14/18, 78%) were multicentre [21, 22, 23, 24, 25, 26, 27, 28, 29, 30, 31, 32, 33, 34] while four were conducted at a single site [35, 36, 37, 38]. Studies were geographically diverse with six originating from the Netherlands [21, 24, 26, 27, 28, 31], two each from China [32, 37], France [33, 34] and the USA [23, 35], and one from each of Australia [22], Sweden [25], Canada [29], Germany [30], Italy [36] and Egypt [38]. Although most studies (15/18, 85%) were registered in a clinical trials registry [21, 23, 24, 26, 27, 28, 29, 30, 31, 33, 34, 35, 36, 37, 38], less than half (8/18, 44%) had a published protocol or published statistical plan [21, 23, 24, 26, 28, 31, 34, 35].

**TABLE 1:** Study characteristics grouped by participant cohort.

First author (year)	Country (centres)	Registered +/protocol published?	Participants	Treatment arms	Primary outcome
			*Patients with UC…*		
McCormick (2012) [19]	Australia (3)	No	Undergoing IPAA	J‐pouch vs. W‐pouch	Bowel function at 1 yr
Vogel (2023) [20]	USA (8)	Yes	Undergoing IPAA	Early ileostomy closure (at 7–12 days) vs. late (≥8 weeks)	CCI
Sapci (2023) [21]	USA (1)	Yes	Undergoing laparoscopic total colectomy	3D laparoscopy vs. 2D laparoscopy	Operative time
The Accure Study Group (2025) [22]	Netherlands (22)	Yes	In remission but treated for relapse within the preceding twelve months	Laparoscopic appendicectomy + standard medical therapy vs. standard medical therapy	Disease relapse in 1 yr
			*Patients with CD…*		
Gerdin (2016) [23]	Sweden (7)	No	With isolated ileocecal disease	Medical treatment vs. ileal or ileocaecal resection	CDAI AUC at 1 yr
Ponsioen (2017) [24]	Netherlands (29)	Yes	Treatment refractory ileocolic disease	Laparoscopic ileocaecal resection vs. infliximab	IBDQ score at 12 mo.
Maartense (2006) [25]	Netherlands (3)	Yes	Undergoing ileocolic resection	Laparoscopic‐assisted vs. open surgical resection	SF‐36 scores at 2‐4 wks
McLeod (2009) [26]	Canada (17)	Yes	Undergoing ileocolic resection	Handsewn end‐to‐end vs. stapled side‐to‐side anastomosis	Endoscopic recurrence at 12 mo.
Zurbuchen (2013) [27]	Germany (9)	Yes	Undergoing ileocolic resection	Handsewn end‐to‐end vs. stapled side‐to‐side anastomosis	Endoscopic recurrence
Luglio (2020) [28]	Italy (1)	Yes	Undergoing ileocolic resection	Kono‐S vs. conventional side‐to‐side anastomosis	Endoscopic recurrence at 6 mo.
Van der Does de Willebois (2024) [29]	Netherlands (6)	Yes	Undergoing ileocolic resection	Extended vs. conventional mesenteric sparing resection	Endoscopic recurrence at 6 mo.
Duan (2025) [30]	China (3)	No	Undergoing ileocolic resection	Limited vs. extensive mesenteric excision	Endoscopic recurrence at 6‐18 mo.
Duan (2024) [35]	China (1)	Yes	Undergoing resection and anastomosis	Prophylactic surgical drain vs. no drain	Postoperative prolonged ileus
Senejoux (2016) [31]	France (16)	Yes	With perianal fistula	Anal fistula plug insertion vs. seton removal	Clinical remission at 12 wks
Abramowitz (2022) [32]	France (11)	Yes	With perianal fistula	Seton removal + surgical closure vs. seton removal	Fistula closure at 12 mo.
Wasmann (2020) [33]	Netherlands (19)	Yes	With perianal fistula	Seton for 1 year vs. anti‐TNF for 1 year vs. surgical closure after 2 months + short course of anti‐TNF	Fistula‐related re‐intervention at 1.5 yrs
Meima‐van Praag (2022) [34]	Netherlands (10)	Yes	With perianal fistula	Short‐term anti‐TNF + surgical closure vs. anti‐TNF	Radiological healing on MRI at 18 mo.
			*Patients with IBD…*		
Zakaria (2025) [36]	Egypt (1)	Yes	With haemorrhoids	Laser haemorrhoidoplasty vs. traditional haemorrhoidectomy	Postoperative pain

Abbreviations: AUC, area under the curve; CCI, Comprehensive Complications Index; CDAI, Crohn's Disease Activity Index; IBDQ, Inflammatory Bowel Disease Questionnaire; IPAA, ileal pouch‐anal anastomosis; SF‐36, Short Form Survey.

**FIGURE 2:**
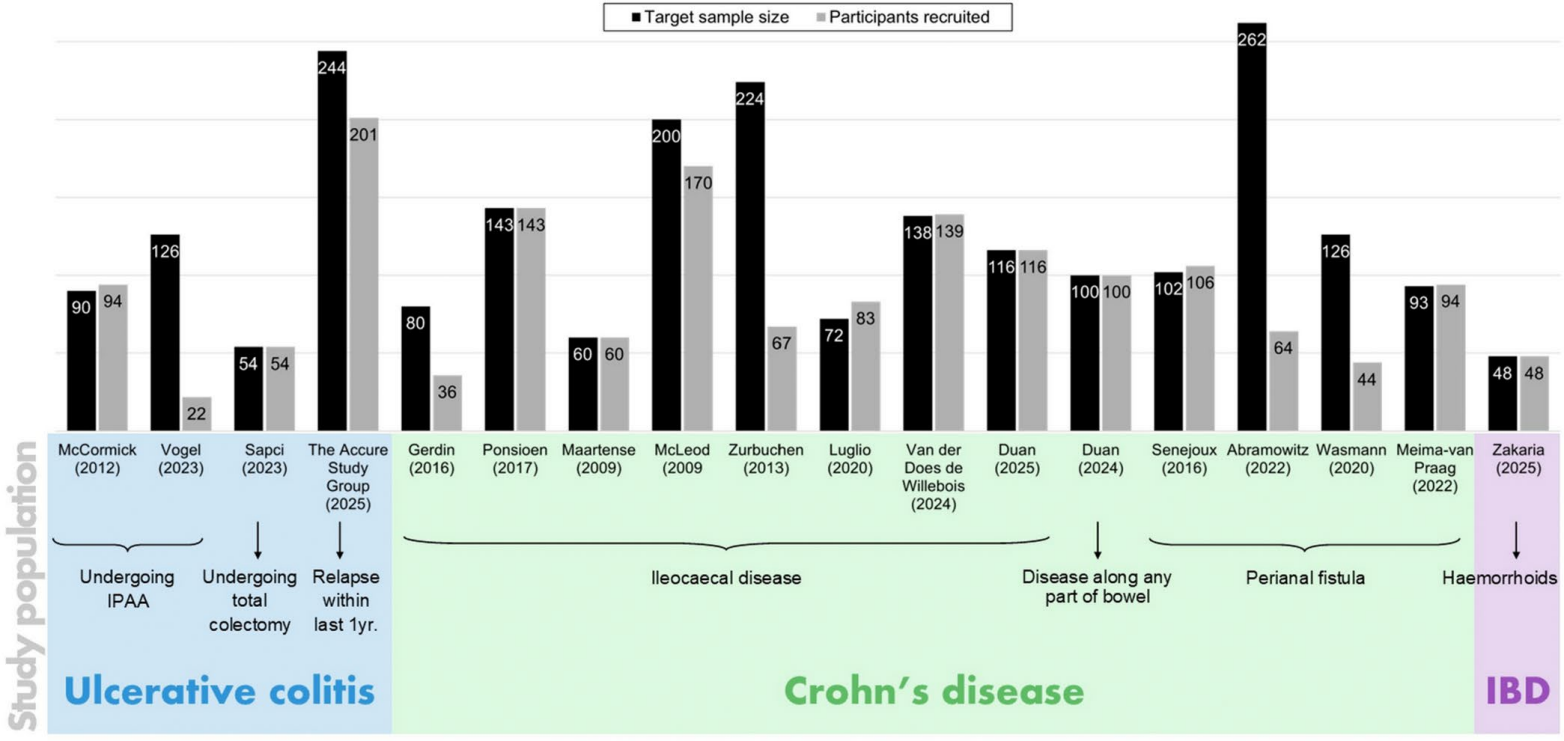
Target sample sies (grey bars) versus actual participant recruitment (black bars) for included RCTs grouped by disease type (blue—Crohn's disease; green—ulcerative colitis; purple—IBD mixed population).

Across the 18 RCTs analysed, most were conducted in patients with CD (13/18, 72%), with fewer focusing on UC (4/18, 22%) or IBD more broadly (1/18, 6%) (Figure 2). Within the 13 CD trials, two specific subgroups were commonly represented: Eight studies (62%) focused on patients with ileocaecal disease undergoing surgical resection and four (31%) focused on perianal fistulating disease. Among the four UC trials, two focused on patients undergoing ileal pouch‐anal anastomosis. The single trial including both CD and UC patients investigated haemorrhoidal disease in individuals with IBD.

There was considerable variability in the choice of primary outcome measures both across and within disease groups. Most trials (11/18, 61%) employed a dichotomous clinical outcome such as disease relapse, endoscopic recurrence, fistula closure and fistula‐related re‐intervention. The remaining trials (7/18, 39%) used continuous outcome measures, which included operating time and validated patient‐reported outcome measures (PROMs) such as the Inflammatory Bowel Disease Questionnaire (IBDQ) and the 36‐item short‐form health survey (SF‐36).

### Trial design and statistical outcomes

Statistical planning and outcomes across the included trials are presented in Table 2. Planned statistical power was declared in 16 of 18 RCTs, with 80% power specified in 12 studies and 90% in four. Effect size assumptions were generally large, with most trials powered to detect absolute differences of 20%–30% or moderate standardised effects (e.g. 0.5). The median intended sample size was 102 participants (interquartile range [IQR] 80–143). Analysis of disease subgroups demonstrated a higher mean intended sample size in CD studies (121 [95–142]) versus UC studies (54 [51–72]). Actual recruitment fell short in many trials, with a median of 89 (IQR 55–114) participants randomised. Again, disease subgroup analysis showed a higher mean enrolment in CD studies (93 [65–114]) than in UC studies (51 [42–64]). Eleven trials (11/18, 61%) recruited to their planned sample size.

**TABLE 2:** Statistical planning and observed outcomes of included RCTs.

First author (year)	Planned statistical power	Assumed effect size (as reported)	Intended sample size	Total participants randomised	Early termination	Loss to follow‐up	Statistically significant primary outcome?
*RCTs in patients with UC*							
McCormick (2012) [19]	80%	1 extra bowel movement per 24 h	90	94	No	14	Yes
Vogel (2023) [20]	80%	Moderate standardised effect size of 0.5	126–152	22	Yes	0	Yes
Sapci (2023) [21]	80%	Not stated	54	54	No	1	No
The Accure Study Group (2025) [22]	90%	20% absolute	244	201	No	4	Yes
*RCTs in patients with CD*							
Gerdin (2016) [23]	80%	50‐point difference	80	36	Yes	13	No
Ponsioen (2017) [24]	80%	An effect size of 0.5	143	143	No	0	No
Maartense (2006) [25]	90%	20‐point difference	60	60	No	8	No
McLeod (2009) [26]	80%	20% absolute	200	170	Yes	31	No
Zurbuchen (2013) [27]	80%	30% absolute	224	67	Yes	67^a^	N/A^a^
Luglio (2020) [28]	90%	30% absolute	72	83	No	4	Yes
Van der Does de Willebois (2024) [29]	80%	25% absolute	138	139	No	8	No
Duan (2025) [30]	None stated	20% absolute	116	116	No	31	Yes
Duan (2024) [35]	None stated	Not stated	100	100	No	0	Yes
Senejoux (2016) [31]	90%	30% absolute	102	106	No	7	No
Abramowitz (2022) [32]	80%	25% relative	262	64	No	6	No
Wasmann (2020) [33]	80%	30% absolute	126	44	Yes	0	Yes
Meima‐van Praag (2022) [34]	80%	30% absolute	93	94	No	0	Yes
*RCTs in patients with IBD*							
Zakaria (2025) [36]	80%	2.6‐point difference	48	48	No	0	Yes

^a^ Primary outcome not assessed due to early termination of the trial.

Five trials (5/18, 28%) were prematurely terminated, with reported reasons including excessive complications on one treatment arm [23], slow recruitment [25, 29], futility [21], overlap with a concurrent trial [30] and evolving clinical practice [25, 30]. Four of these studies were conducted in CD populations and one in UC. Loss to follow‐up averaged 7 participants per study (range 0–31). Notably, in one study, no participants were analysed for the primary outcome due to early termination of the trial. Half of the studies (9/18) reported a statistically significant primary outcome, including two that had either failed to reach the enrolment target or had been prematurely stopped.

### Assessment of fragility and trial pragmatism

**FIGURE 3:**
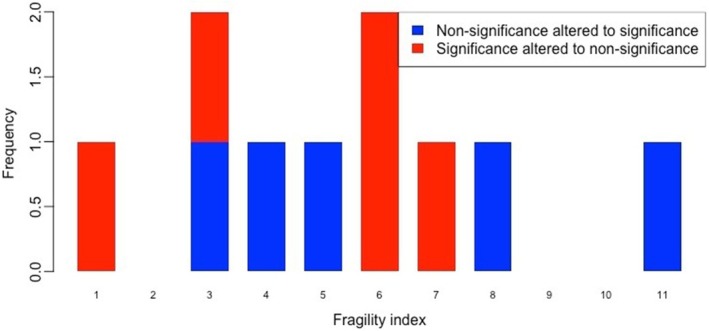
Bar graph demonstrating fragility index (red bars) and reverse fragility index (blue bars).

Ten RCTs employing a two‐group comparison design and a dichotomous primary endpoint were eligible for analysis of statistical robustness. Five studies had a statistically significant between‐groups difference in the primary outcome and underwent Fragility Index analysis; the remaining five studies, which demonstrated a non‐statistically significant difference in the primary outcome measure, were analysed for Reverse Fragility Index (Figure 3). The median (IQR) FI for RCTs with statistically significant primary outcomes was 3 (IQR 1–6), and the RFI for RCTs with non‐statistically significant primary outcomes was 4 (IQR 3–8).

**FIGURE 4:**
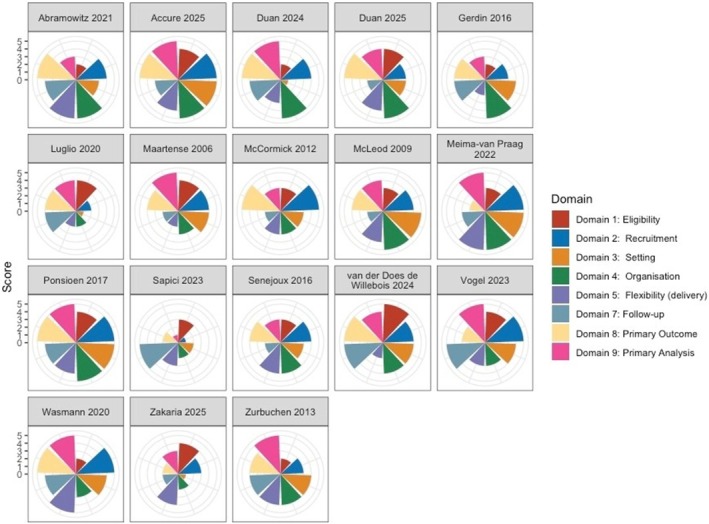
PRECIS‐2 wheels for included RCTs.

The PRECIS‐2 scores for each study are presented in Figure 4. PRECIS‐2 assessment showed a tendency towards pragmatic designs. Mean scores were consistent across domains, ranging narrowly from 3.2 to 4.1.

### Assessment of risk of bias

**FIGURE 5:**
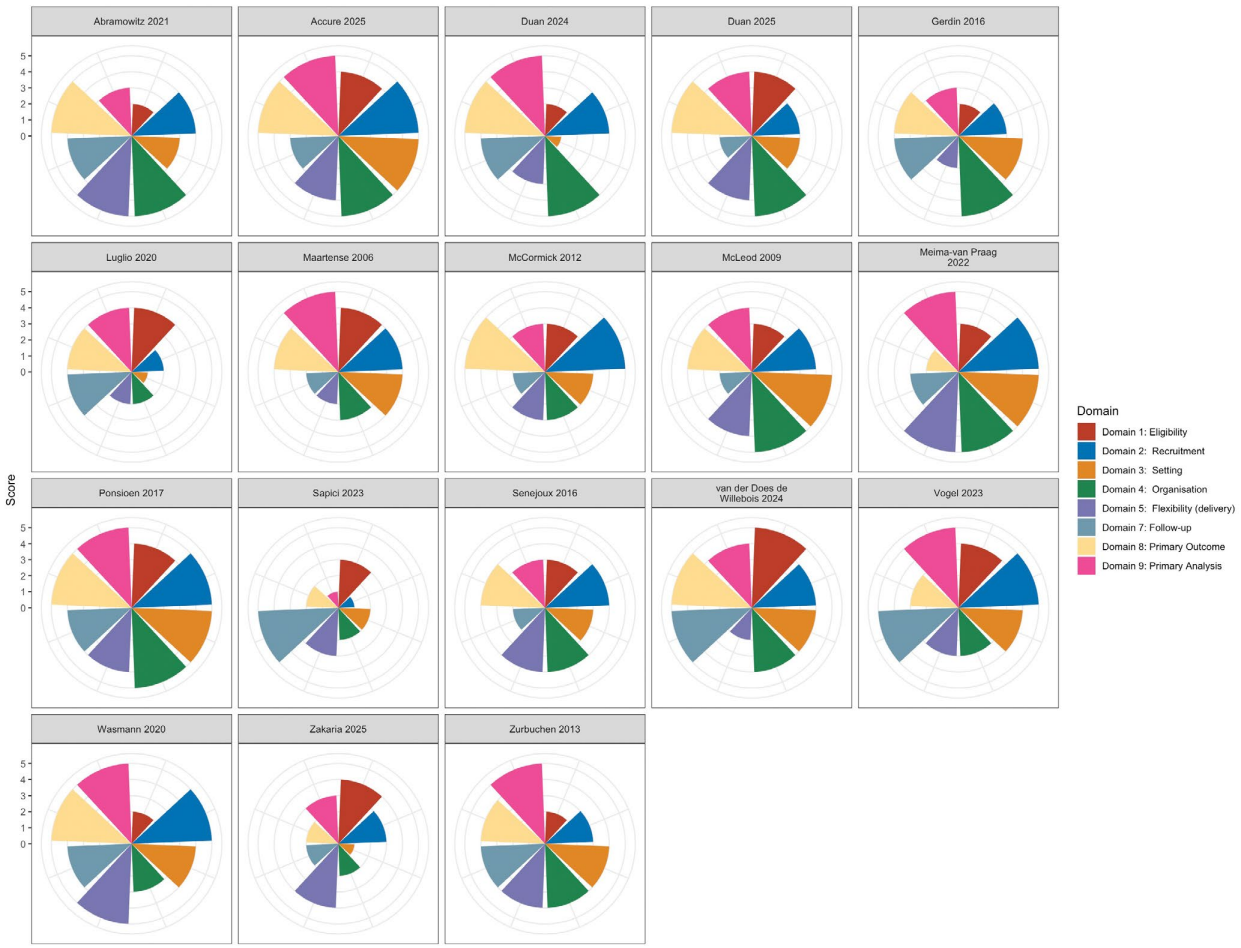
Cochrane Risk of Bias assessment. (A) Summary of the risk of bias for each domain and the overall assessment for included RCTs. (B) Bar chart summarising the proportions of risk of bias across domains.

Risk of bias was assessed in all included RCTs and is reported by domain in Figure 5. Most studies (12/18, 67%) had some concern for bias in one or more domains. Five studies (28%) had low risk of bias, and no studies had high risk of bias. Assessment could not be completed across all domains for one study which was terminated prematurely and did not analyse the stated primary outcome [30].

### Unpublished registered clinical trials

The study characteristics of the 26 identified registered clinical trials are summarised in Table S1. Of the 26 registered trials, the vast majority were focused on CD patients (85%, 22/26) compared to just four trials in UC patients. Three predominant subgroups were apparent within the CD trials: ileocaecal disease (17/22), stricturing disease (4/22) and perianal fistulising disease (1/22). The median intended sample size was 155 (IQR 93–196). Classified by disease subgroups, the median intended sample size was higher for CD trials than for UC trials (162 [117–201] and 89 [75–119], respectively).

## DISCUSSION

This systematic review provides an overview of the current landscape of RCTs in IBD surgery, identifying common features of trial design and highlighting recurring challenges in trial delivery. While pragmatic designs predominated, trials were frequently constrained by modest planned sample sizes, under‐recruitment and high rates of early termination, with consequent risks around statistical fragility.

This manuscript has focused on surgical RCTs. These are fundamentally different studies to cohort studies and perform different roles. A cohort study provides large volumes of evidence around variation and supports hypothesis generation. An RCT seeks to demonstrate a causal link between intervention and outcome. Cohort studies in surgery are not beset by the same challenges as RCTs. For example, the recent DAMASCUS cohort study gathered data on more than 6000 patients, from 248 hospitals in 48 countries. This is the level of collaboration and efficiency we should be seeking in IBD surgery.

Sample sizes across the IBD surgical trials were broadly limited, with a median of 89 participants recruited. Even among contemporarily registered RCTs, we found that large‐scale enrolment remains uncommon, with only one in five designed to recruit more than 200 participants, the largest aiming for 550. These figures are consistent with previous reports of constrained sample sizes in surgical RCTs [39, 40]. It is notable that statistical simulations suggest that trials with fewer than 100 participants are useful as pilot trials to inform sample size estimations in a full RCT, rather than being definitive in their own right [41]. In principle, sample size calculations are based on effect size assumptions and desired statistical power; however, practical considerations such as resource constraints often mean that recruitment is limited to the minimum number of participants required to meet the calculated threshold. Surgical RCTs designed to detect the minimum difference deemed clinically and statistically relevant are inherently vulnerable to statistical fragility. A median fragility index of 3 indicates that many statistically significant findings in IBD surgical RCTs depend on very small numbers of outcome events and should therefore be interpreted with caution when informing clinical practice.

Review of sample size assumptions revealed that many trials were designed to detect relatively large treatment effects. Across studies, commonly assumed effect sizes included absolute differences of 20%–30% in dichotomous outcomes and standardised effect sizes of 0.5 for continuous measures. While such assumptions may be appropriate in early‐phase or exploratory studies, they are optimistic in the context of complex surgical interventions where treatment effects are often more modest. In several cases, these ambitious assumptions were coupled with under‐recruitment or early termination, further reducing statistical power. This combination of optimistic effect size assumptions and incomplete recruitment likely contributes to the statistical fragility observed across trials, and raises concerns that many studies were not realistically powered to detect clinically meaningful differences.

This potential vulnerability may be further exacerbated by under‐recruitment and early termination, both of which were evident in our review and are well‐recognised challenges in surgical research [42, 43]. Recruitment shortfalls were common, with 40% of RCTs not reaching their planned sample size—a proportion comparable with prior reports that this is experienced in around one in three surgical trials [42]. Early termination occurred in 28% of RCTs, again echoing previously reported rates of around 20%, with reasons reflective of known barriers in the field including slow accrual, adverse events and concurrent studies [43]. Stopping for futility also emerged as a recurring theme, highlighting the difficulty of sustaining equipoise when emerging data or changing standards of care challenge the original trial hypothesis. These issues underscore the importance of careful feasibility and pilot work, alongside robust patient and public involvement (PPI) in the design and delivery of future trials, to ensure that questions remain relevant and recruitment strategies acceptable. It is also notable that despite the challenges of recruitment in IBD surgery, a proportion of trials continue to be conducted at single centres, further limiting generalisability and scale. Collectively, these challenges raise concerns that many surgical RCTs remain underpowered, undermining the reliability of their conclusions and heightening the risk that meaningful treatment effects go unrecognised.

The PRECIS‐2 analysis demonstrated that most IBD surgical RCTs sit towards the pragmatic end of the trial design spectrum, reflecting their multicentre recruitment and broad eligibility criteria. While this approach enhances real‐world applicability, it does not in itself guarantee external validity. Ensuring adequate sample size, sufficient statistical power and careful consideration of how findings translate beyond the study setting remain essential. On the contrary, more explanatory designs may still be appropriate for mechanistic questions (e.g. optimal anastomotic technique), but such trials should be clearly framed as exploratory. Ultimately, to influence practice and guidelines, future IBD surgical RCTs will need to be pragmatic, multicentre and sufficiently resourced.

The validated tools used to assess IBD surgical trials in this review—FI, PRECIS‐2 and RoB 2—have been widely used to analyse RCTs but do possess notable limitations. As the FI is restricted to dichotomous variables in two‐arm trials, all RCTs utilising continuous primary endpoints or more than two treatment arms were excluded from our analysis of statistical robustness. The considerable variation in primary outcomes and trial methodologies, which limited the use of FI analysis, reflected broader heterogeneity across the RCTs in surgical interventions and patient subgroups, making direct comparisons and generalisation difficult. The PRECIS‐2 and RoB 2 tools rely on subjective ratings by reviewers, often requiring nuanced judgements on sometimes incomplete or ambiguous information, especially when applied to trials retrospectively. PRECIS‐2 was originally developed to guide trial design at the planning stage; however, there is precedence for retrospective application of this tool to assess pragmatic intentions [39, 44, 45]. Importantly, differences in inter‐rater reliability for PRECIS‐2 have been highlighted [46]. To mitigate the influence of individual bias in this review, PRECIS‐2 and RoB 2 assessments were performed independently by two researchers, with a third reviewer resolving any discrepancies. This rigorous approach was applied to all critical stages—screening, data extraction and analysis—thereby enhancing reliability and reducing errors. A further strength of this study is the consideration of unpublished registered RCTs in addition to published trials, providing a more comprehensive view of IBD surgical research.

Based on these findings, we propose a set of practical recommendations aimed at colorectal surgeons, collaborative research groups and funders to improve the design and delivery of future IBD surgical trials (Table 3). To advance the field, future IBD surgical RCTs must be larger, build on strong collaborations and be transparently conducted, with core outcomes and rigorous design tools applied prospectively. Furthermore, research funders should expect and support ambitious sample sizes which will provide definitive answers to research questions. This will require infrastructure to support such research across geographic borders, and realistic funding envelopes to deliver this. Implementing the recommendations outlined in this review will be crucial for researchers, funders and commissioners seeking to generate robust, practice‐changing evidence to guide surgical care in IBD.

**TABLE 3:** Summary of recommendations for future trials.

Identified problem	Observed pattern in current trials	Implications for validity and impact	Recommended design and delivery solutions
Small planned sample sizes	Median planned sample size ~100 participants; few trials >200	Trials powered for optimistic effect sizes; high risk of false‐negative results	Design around realistic effect sizes; treat trials <100 participants as pilot/feasibility unless strongly justified
Under‐recruitment	~40% of trials fail to reach recruitment targets	Loss of power; compromised credibility of negative findings	Mandatory internal pilot phases with stop–go criteria; active recruitment monitoring
Early trial termination	Nearly one‐third stopped early due to slow accrual, futility or evolving practice	Reduced statistical reliability; increased fragility; limited interpretability	Robust feasibility work; adaptive designs where appropriate; clear equipoise justification
Statistical fragility of positive results	Median fragility index of 3 among statistically significant trials	Positive findings hinge on small numbers of events	Pre‐specify fragility assessments; avoid over‐interpretation of marginal significance
Heterogeneous primary outcomes	Wide variation in endpoints across disease subgroups	Limits cross‐trial comparison and cumulative evidence synthesis	Develop and mandate core outcome sets including PROMs
Limited protocol transparency	Less than half of trials publish protocols or SAPs	Increased risk of selective reporting and reduced reproducibility	Require protocol and SAP publication prior to recruitment
Single‐centre or small network designs	Ongoing reliance on single‐centre or low‐volume multicentre trials	Restricted generalisability and inefficient recruitment	Use national and international collaborative trial platforms
Mismatch between pragmatic intent and power	Trials score pragmatic on PRECIS‐2 but remain underpowered	Real‐world relevance without reliability limits practice change	Align pragmatic design with sufficient sample size and infrastructure
Limited funder ambition for scale	Trials designed to minimum viable funding envelopes	Cycle of small, fragile studies with limited impact	Funders to prioritise fewer, larger, definitive trials

## AUTHOR CONTRIBUTIONS

**Kelsey Aimar:** Conceptualization; methodology; investigation; writing – original draft; writing – review and editing; data curation; formal analysis; validation; project administration. **Aditya Gaur:** Writing – review and editing; data curation; validation; investigation; writing – original draft. **Thomas D. Pinkney:** Writing – review and editing; investigation; conceptualization; methodology; validation; formal analysis; supervision. **Michael Peirson:** Investigation; methodology; data curation; writing – review and editing; writing – original draft. **Josephine Walshaw:** Conceptualization; investigation; validation; methodology; formal analysis; data curation; writing – review and editing; writing – original draft. **Matthew J. Lee:** Conceptualization; investigation; writing – original draft; writing – review and editing; visualization; methodology; supervision; formal analysis; resources.

## FUNDING INFORMATION

No funding received.

## CONFLICT OF INTEREST STATEMENT

The authors declare no conflicts of interest.

## ETHICS STATEMENT

None.

## Supporting information


**Data S1:** Supporting Information

**Data S2:** Supporting Information

## Data Availability

Data sharing not applicable to this article as no datasets were generated or analysed during the current study.

## References

[1] Tsai L , Ma C , Dulai PS , Prokop LJ , Eisenstein S , Ramamoorthy SL , et al. Contemporary risk of surgery in patients with ulcerative colitis and Crohn's disease: a meta‐analysis of population‐based cohorts. Clin Gastroenterol Hepatol. 2021;19(10):2031–2045.e11. 33127595 10.1016/j.cgh.2020.10.039 PMC8934200

[2] Frolkis AD , Dykeman J , Negrón ME , Debruyn J , Jette N , Fiest KM , et al. Risk of surgery for inflammatory bowel diseases has decreased over time: a systematic review and meta‐analysis of population‐based studies. Gastroenterology. 2013;145(5):996–1006. 23896172 10.1053/j.gastro.2013.07.041

[3] Parragi L , Fournier N , Zeitz J , Scharl M , Greuter T , Schreiner P , et al. Colectomy rates in ulcerative colitis are low and decreasing: 10‐year follow‐up data from the Swiss IBD cohort study. J Crohns Colitis. 2018;12(7):811–818. 29617750 10.1093/ecco-jcc/jjy040

[4] Targownik LE , Singh H , Nugent Z , Bernstein CN . The epidemiology of colectomy in ulcerative colitis: results from a population‐based cohort. Am J Gastroenterol. 2012;107(8):1228–1235. 22613902 10.1038/ajg.2012.127

[5] Adamina M , Minozzi S , Warusavitarne J , Buskens CJ , Chaparro M , Verstockt B , et al. ECCO guidelines on therapeutics in Crohn's disease: surgical treatment. J Crohns Colitis. 2024;18(10):1556–1582. 38878002 10.1093/ecco-jcc/jjae089

[6] Search ‐ NIHR funding and awards [Internet]. [cited 2026 Jan 21]. Available from: https://fundingawards.nihr.ac.uk/award/NIHR131988

[7] Hart AL , Lomer M , Verjee A , Kemp K , Faiz O , Daly A , et al. What are the top 10 research questions in the treatment of inflammatory bowel disease? A priority setting partnership with the James Lind Alliance. J Crohns Colitis. 2017;11(2):204–211. 27506537 10.1093/ecco-jcc/jjw144 PMC5266081

[8] Bothwell LE , Greene JA , Podolsky SH , Jones DS . Assessing the gold standard—lessons from the history of RCTs. N Engl J Med. 2016;374(22):2175–2181. 27248626 10.1056/NEJMms1604593

[9] Ma C , Jairath V , Feagan BG , Peyrin‐Biroulet L , Danese S , Sands BE , et al. Interpreting modern randomized controlled trials of medical therapy in inflammatory bowel disease. Nat Rev Gastroenterol Hepatol. 2024;21(11):792–808. 39379665 10.1038/s41575-024-00989-y

[10] Cai Z , Wang S , Li J . Treatment of inflammatory bowel disease: a comprehensive review. Front Med (Lausanne). 2021;8:765474. 34988090 10.3389/fmed.2021.765474 PMC8720971

[11] Catt H , Hughes D , Kirkham JJ , Bodger K . Systematic review: outcomes and adverse events from randomised trials in Crohn's disease. Aliment Pharmacol Ther. 2019;49(8):978–996. 30828852 10.1111/apt.15174 PMC6492112

[12] Moreira PL , Dignass A , Estevinho MM , Portal F , Mendes J , Santiago M , et al. Assessment of outcomes in Crohn's disease: a systematic review of randomized clinical trials to inform a multiple outcome framework. United European Gastroenterol J. 2024;12(9):1280–1291. 10.1002/ueg2.12679 PMC11578837 39391955

[13] Walsh M , Srinathan SK , McAuley DF , Mrkobrada M , Levine O , Ribic C , et al. The statistical significance of randomized controlled trial results is frequently fragile: a case for a fragility index. J Clin Epidemiol. 2014;67(6):622–628. 24508144 10.1016/j.jclinepi.2013.10.019

[14] Loudon K , Treweek S , Sullivan F , Donnan P , Thorpe KE , Zwarenstein M . The PRECIS‐2 tool: designing trials that are fit for purpose. BMJ. 2015;350(may08 1):h2147. 25956159 10.1136/bmj.h2147

[15] Higgins JPT , Thomas J , Chandler J , Cumpston M , Li T , Page MJ , et al. (eds). Cochrane Handbook for Systematic Reviews of Interventions version 6.5 (updated August 2024). Cochrane, 2024. Available from: https://www.cochrane.org/handbook

[16] PRISMA 2020 checklist—[Internet] . PRISMA statement. [cited 2024 Nov 16]. Available from: https://www.prisma‐statement.org/prisma‐2020‐checklist

[17] Lin L , Chu H . Assessing and visualizing fragility of clinical results with binary outcomes in R using the fragility package. PLoS One. 2022;17(6):e0268754. 35648746 10.1371/journal.pone.0268754 PMC9159630

[18] Computing R, Others . R: a language and environment for statistical computing. Vienna: R Core Team [Internet]; 2013. Available from: https://www.yumpu.com/en/document/view/6853895/r‐a‐language‐and‐environment‐for‐statistical‐computing

[19] Caldwell JME , Youssefzadeh K , Limpisvasti O . A method for calculating the fragility index of continuous outcomes. J Clin Epidemiol. 2021;136:20–25. 33684509 10.1016/j.jclinepi.2021.02.023

[20] RoB 2: A revised Cochrane risk‐of‐bias tool for randomized trials [Internet]. [cited 2023 Dec 29]. Available from: https://methods.cochrane.org/bias/resources/rob‐2‐revised‐cochrane‐risk‐bias‐tool‐randomized‐trials

[21] Wasmann KA , de Groof EJ , Stellingwerf ME , D'Haens GR , Ponsioen CY , Gecse KB , et al. Treatment of perianal fistulas in Crohn's disease, seton versus anti‐TNF versus surgical closure following anti‐TNF [PISA]: a randomised controlled trial. J Crohns Colitis. 2020;14(8):1049–1056. 31919501 10.1093/ecco-jcc/jjaa004 PMC7476637

[22] McCormick PH , Guest GD , Clark AJ , Petersen D , Clark DA , Stevenson AR , et al. The ideal ileal‐pouch design: a long‐term randomized control trial of J‐ vs W‐pouch construction. Dis Colon Rectum. 2012;55(12):1251–1257. 23135583 10.1097/DCR.0b013e318270327f

[23] Vogel JD , Fleshner PR , Holubar SD , Poylin VY , Regenbogen SE , Chapman BC , et al. High complication rate after early ileostomy closure: early termination of the short versus long interval to loop ileostomy reversal after pouch surgery randomized trial. Dis Colon Rectum. 2023;66(2):253–261. 36627253 10.1097/DCR.0000000000002427

[24] ACCURE Study Group . Appendicectomy plus standard medical therapy versus standard medical therapy alone for maintenance of remission in ulcerative colitis (ACCURE): a pragmatic, open‐label, international, randomised trial. Lancet Gastroenterol Hepatol. 2025;10(6):550–561. 40228513 10.1016/S2468-1253(25)00026-3 PMC12062198

[25] Gerdin L , Eriksson AS , Olaison G , Sjödahl R , Ström M , Söderholm JD , et al. The Swedish Crohn trial: a prematurely terminated randomized controlled trial of thiopurines or open surgery for primary treatment of ileocaecal Crohn's disease. J Crohns Colitis. 2016;10(1):50–54. 26507858 10.1093/ecco-jcc/jjv184

[26] Ponsioen CY , de Groof EJ , Eshuis EJ , Gardenbroek TJ , Bossuyt PMM , Hart A , et al. Laparoscopic ileocaecal resection versus infliximab for terminal ileitis in Crohn's disease: a randomised controlled, open‐label, multicentre trial. Lancet Gastroenterol Hepatol. 2017;2(11):785–792. 28838644 10.1016/S2468-1253(17)30248-0

[27] Maartense S , Dunker MS , Slors JFM , Cuesta MA , Pierik EGJM , Gouma DJ , et al. Laparoscopic‐assisted versus open ileocolic resection for Crohn's disease: a randomized trial. Ann Surg. 2006;243(2):143–149; discussion 150–3. 16432345 10.1097/01.sla.0000197318.37459.ec PMC1448907

[28] Meima‐van Praag EM , van Rijn KL , Wasmann KATGM , Snijder HJ , Stoker J , D'Haens GR , et al. Short‐term anti‐TNF therapy with surgical closure versus anti‐TNF therapy in the treatment of perianal fistulas in Crohn's disease (PISA‐II): a patient preference randomised trial. Lancet Gastroenterol Hepatol. 2022;7(7):617–626. 35427495 10.1016/S2468-1253(22)00088-7

[29] McLeod RS , Wolff BG , Ross S , Parkes R , McKenzie M , Investigators of the CAST Trial . Recurrence of Crohn's disease after ileocolic resection is not affected by anastomotic type: results of a multicenter, randomized, controlled trial. Dis Colon Rectum. 2009;52(5):919–927. 19502857 10.1007/DCR.0b013e3181a4fa58

[30] Zurbuchen U , Kroesen AJ , Knebel P , Betzler MH , Becker H , Bruch HP , et al. Complications after end‐to‐end vs. side‐to‐side anastomosis in ileocecal Crohn's disease—early postoperative results from a randomized controlled multi‐center trial (ISRCTN‐45665492). Langenbeck's Arch Surg. 2013;398(3):467–474. 22290216 10.1007/s00423-012-0904-1

[31] van der Does de Willebois EML , Bellato V , Duijvestein M , van der Bilt JDW , van Dongen K , Spinelli A , et al. Effect of mesenteric sparing or extended resection in primary ileocolic resection for Crohn's disease on postoperative endoscopic recurrence (SPICY): an international, randomised controlled trial. Lancet Gastroenterol Hepatol. 2024;9(9):793–801. 39025100 10.1016/S2468-1253(24)00097-9

[32] Duan M , Liu W , Coffey JC , Ke J , Zhou W , Li Y , et al. Postoperative endoscopic outcomes in the MESOCOLIC trial investigating mesenteric‐based surgery for crohn's disease. Gastroenterology. 2025;168(5):987–990.e5. 39798672 10.1053/j.gastro.2024.12.028

[33] Senéjoux A , Siproudhis L , Abramowitz L , Munoz‐Bongrand N , Desseaux K , Bouguen G , et al. Fistula plug in fistulising Ano‐perineal Crohn's disease: a randomised controlled trial. J Crohns Colitis. 2016;10(2):141–148. 26351393 10.1093/ecco-jcc/jjv162

[34] Abramowitz L , Brochard C , Pigot F , Roumeguere P , Pillant H , Vinson Bonnet B , et al. Surgical closure, mainly with glue injection and anti‐tumour necrosis factor α, in fistulizing perianal Crohn's disease: a multicentre randomized controlled trial. Color Dis. 2022;24(2):210–219. 10.1111/codi.15947 34623746

[35] Sapci I , GamalEldin M , Rencuzogullari A , Yilmaz S , Kessler H , Hull T , et al. Prospective randomized comparison of three‐dimensional (3D) versus conventional laparoscopy in total colectomy for ulcerative colitis. ANZ J Surg. 2023;93(9):2155–2160. 36898957 10.1111/ans.18368

[36] Luglio G , Rispo A , Imperatore N , Giglio MC , Amendola A , Tropeano FP , et al. Surgical prevention of anastomotic recurrence by excluding mesentery in crohn's disease: the SuPREMe‐CD study—a randomized clinical trial. Ann Surg. 2020;272(2):210–217. 32675483 10.1097/SLA.0000000000003821

[37] Duan M , Cao L , Lu M , Zhang T , Ji Q , Guo X , et al. Prophylactic intra‐abdominal drainage is associated with lower postoperative complications in patients with Crohn's disease: a randomized controlled trial. Surg Innov. 2024;31(2):157–166. 38339842 10.1177/15533506241232598

[38] Zakaria R , Amin MM , Abo‐Alella HA , Hegab YH . Laser hemorrhoidoplasty versus hemorrhoidectomy in the treatment of surgically indicated hemorrhoids in inflammatory bowel patients: a randomized comparative clinical study. Surg Endosc. 2025;39(1):249–258. 39511001 10.1007/s00464-024-11351-3 PMC11666749

[39] Robinson NB , Fremes S , Hameed I , Rahouma M , Weidenmann V , Demetres M , et al. Characteristics of randomized clinical trials in surgery from 2008 to 2020: a systematic review. JAMA Netw Open. 2021;4(6):e2114494. 34190996 10.1001/jamanetworkopen.2021.14494 PMC8246313

[40] Ahmed Ali U , Ten Hove JR , Reiber BM , van der Sluis PC , Besselink MG . Sample size of surgical randomized controlled trials: a lack of improvement over time. J Surg Res. 2018;228:1–7. 29907196 10.1016/j.jss.2018.02.014

[41] Teare MD , Dimairo M , Shephard N , Hayman A , Whitehead A , Walters SJ . Sample size requirements to estimate key design parameters from external pilot randomised controlled trials: a simulation study. Trials. 2014;15(1):264. 24993581 10.1186/1745-6215-15-264 PMC4227298

[42] Cade Shadbolt MA , Elise Naufal B , Siddharth Rele B , Schilling C , Sharmala Thuraisingam M , Stefan Lohmander L , et al. Analysis of rates of completion, delays, and participant recruitment in randomized clinical trials in surgery. JAMA Netw Open. 2023;6(1):e2250996. 36648945 10.1001/jamanetworkopen.2022.50996 PMC9857498

[43] Wartolowska K , Collins GS , Hopewell S , Judge A , Dean BJF , Rombach I , et al. Feasibility of surgical randomised controlled trials with a placebo arm: a systematic review. BMJ Open. 2016;6(3):e010194. 10.1136/bmjopen-2015-010194 PMC4800115 27008687

[44] Dal‐Ré R , Janiaud P , Ioannidis JPA . Real‐world evidence: how pragmatic are randomized controlled trials labeled as pragmatic? BMC Med. 2018;16(1):49. 29615035 10.1186/s12916-018-1038-2 PMC5883397

[45] Hohenschurz‐Schmidt D , Kleykamp BA , Draper‐Rodi J , Vollert J , Chan J , Ferguson M , et al. Pragmatic trials of pain therapies: a systematic review of methods. Pain. 2022;163(1):21–46. 34490854 10.1097/j.pain.0000000000002317 PMC8675058

[46] Neta G , Johnson KE . Informing real‐world practice with real‐world evidence: the value of PRECIS‐2. BMC Med. 2018;16(1):76. 29783964 10.1186/s12916-018-1071-1 PMC5963183

