# Supplementary material for: A systematic review of the methodological quality of randomised trials in IBD surgery
Source: Colorectal Dis. 2026 Jun 18;28(6):e70531. doi: 10.1111/codi.70531 (PMC13280174; doi:10.1111/codi.70531)
Supplement: Supplementary file 1 — Data S1: Supporting Information [file CODI-28-0-s002.docx]

**Supplementary Materials - Index**

| **Supplementary Methods** |  |
| --- | --- |
| Search Strategy | *Page 2* |
| **Supplementary Tables** |  |
| Supplementary Table | *Page 3* |
| **References** | *Page 5* |
|  |  |

**Search Strategy**

| **Database:** | **Cochrane Central Register of Controlled Trials (CENTRAL) and Cochrane Database of Systematic Reviews (CDSR)** | **Results per line:** |
| --- | --- | --- |
| **Date:** | **22/05/2025** |  |
| #1 | MeSH descriptor: [Inflammatory Bowel Diseases] explode all trees | 5035 |
| #2 | MeSH descriptor: [Colitis] explode all trees | 2536 |
| #3 | MeSH descriptor: [Enteritis] explode all trees | 312 |
| #4 | MeSH descriptor: [Enterocolitis] this term only | 136 |
| #5 | MeSH descriptor: [Enterocolitis, Necrotizing] this term only | 429 |
| #6 | MeSH descriptor: [Proctitis] this term only | 158 |
| #7 | MeSH descriptor: [Megacolon, Toxic] this term only | 2 |
| #8 | ((inflamm* NEAR/2 (colon* or bowel*))):ti,ab,kw | 5090 |
| #9 | ((ulcer* NEAR/2 colitis)):ti,ab,kw | 7141 |
| #10 | ((IBD or crohn*)):ti,ab,kw | 8103 |
| #11 | ((colitis or colitide* or colorectitis or cuffitis or duodenitis or enteritis or enterocolitis or ileitis or megacolon or pancolitis or proctitis or proctocolitis or pouchitis or proctosigmoiditis or rectocolitis or rectosigmoiditis)):ti,ab,kw | 12866 |
| #12 | ((epithelioid NEAR/2 granuloma*)):ti,ab,kw | 6 |
| #13 | {OR #1-#12} | 19541 |
| #14 | MeSH descriptor: [Digestive System Surgical Procedures] this term only | 1052 |
| #15 | MeSH descriptor: [Enterostomy] explode all trees | 640 |
| #16 | MeSH descriptor: [Surgical Procedures, Colorectal] explode all trees | 1140 |
| #17 | MeSH descriptor: [Colonic Pouches] this term only | 72 |
| #18 | ((surger* or surgical* or operat*)):ti | 114993 |
| #19 | (((bowel or colon* or intestin* or ileo* or ileal* or rectal* or rectum*) NEAR/3 resect*)):ti,ab,kw | 3823 |
| #20 | (((ileo* or ileal*) NEAR/3 anastomosis)):ti,ab,kw | 416 |
| #21 | (((colon* or j or ileo* or ileal*) NEAR/2 (pouch* or reservoir*))):ti,ab,kw | 378 |
| #22 | ((cecostom* or colostom* or colectom* or proctocolectom* or rectocolectom* or hemicolectom* or ileostom* or enterostom* or enterectom* or fistulotom* or strictureplast* or stricturoplast* or IPAA)):ti,ab,kw | 4306 |
| #23 | {OR #14-#22} | 120930 |
| #24 | #13 AND #23 with Publication Year from 2005 to 2025, in Trials | 1322 |

**Supplementary Table. Study characteristics of unpublished registered clinical trials, stratified by study participants and estimated date of study completion.**

| **Country**  **Trial registration** | **Participants**  **(intended sample size)** | **Treatment arms** | | **Recruitment status** | **Estimated date study completion** |
| --- | --- | --- | --- | --- | --- |
|  | *Patients with UC…* |  | | | |
| China  NCT03536988^1^ | …undergoing proctectomy and IPAA (84) | Transanal vs. transabdominal proctectomy with IPAA | | Unknown | Dec 2020 |
| France  NCT03872271^2^ | …or IBDU undergoing proctectomy and IPAA (194) | Without diverting loop ileostomy vs. with diverting loop ileostomy | | Unknown | May 2025 |
| Canada  NCT04722757^3^ | …or IBDU with disease refractory to therapy or with dysplasia (48) | Transanal vs. transabdominal IPAA | | Recruiting | June 2025 |
| Italy  NCT05931458^4^ | …with left-sided disease refractory to therapy (94) | Laparoscopic appendicectomy vs. infliximab | | Not yet recruiting | July 2028 |
|  | *Patients with stricturing CD…* | |  | | |
| Canada  NCT03735355^5^ | …with a short stricture within reach of endoscopy (40) | Ballon dilatation vs. surgical resection | | Unknown | Feb 2022 |
| China  NCT04865484^6^ | …with multi-segmental intestinal strictures (120) | Surgical resection + endoscopic stricturotomy vs. surgical resection + strictureplasty | | Unknown | Dec 2023 |
| France  NCT05584228^7^ | …with intestinal stricture(s) responsible for obstructive symptoms (150) | Medical therapy (azathioprine + infliximab) vs. surgical resection | | Not yet recruiting | Oct 2027 |
| China  NCT05421455^8^ | …with intestinal stricture(s) responsible for obstructive symptoms (138) | Medical treatment with biologics vs. surgical treatment | | Recruiting | June 2030 |
|  | *Patients with ileocolic CD…* |  | | | |
| Netherlands  NL-OMON31330^9^ | …with recurrent terminal ileitis (130) | Laparoscopic ileocolic resection vs. infliximab | | Pending | Unknown |
| China  ChiCTR-2000029323^10^ | (106) | Laparoscopic bowel resection + infliximab vs. infliximab | | Pending | Unknown |
| Czechia  NCT02716454^11^ | (200) | Early laparoscopic ileocolic resection vs. step-up conservative approach | | Unknown | Dec 2021 |
|  | *Patients with CD undergoing ileocolic resection…* |  | | | |
| USA  NCT03172143^12^ | (target sample size not stated) | High ligation of ileocolic artery vs. mesenteric sparing | | Terminated | Terminated: Accrual Not Met |
| UK  NCT01876264^13^ | (target sample size not stated) | Extended vs. conventional ileocolic resection | | Withdrawn | Study abandoned |
| China  ChiCTR-  INR-16008585^14^ | (159) | End-to-side vs. unprecisely side-to-side vs. precisely side-to-side anastomosis | | Pending | Unknown |
| China  ChiCTR-2000035737^15^ | (90) | Resection determined by indocyanine green fluorescence angiography vs. 2cm margin | | Pending | Unknown |
| Italy  NCT05246917^16^ | (189) | Handsewn (Kono-S or end-to-end) vs. stapled side-to-side anastomosis | | Unknown | Dec 2024 |
| UK  ISRCTN16900055^17^ | (308) | Kono-S + radical mesenteric resection vs. Kono-S + close mesenteric resection vs. standard anastomosis + radical mesenteric resection vs. standard anastomosis + close mesenteric resection | | No longer recruiting | April 2026 |
| Netherlands  NCT05578235^18^ | (165) | Handsewn vs. stapled side-to-side anastomosis | | Recruiting | Oct 2027 |
| USA  NCT04578392^19^ | (181) | High ligation of ileocolic artery vs. mesenteric sparing | | Recruiting | Dec 2027 |
| France  NCT05974358^20^ | (226) | Kono-S vs. conventional side-to-side anastomosis | | Recruiting | Feb 2029 |
| Denmark  NCT06324838^21^ | (204) | Extended vs. standard mesenteric resection | | Not yet recruiting | June 2029 |
| China  NCT05658081^22^ | (236) | Stapled Kono-S vs. stapled side-to-side anastomosis | | Recruiting | Dec 2029 |
| Australia  U1111-1276-0767^23^ | (65) | Modified Kono-S vs. stapled side-to-side anastomosis | | Recruiting | Dec 2031 |
| China  NCT06241170^24^ | (172) | Mesentery-guided vs. traditional resection margin | | Recruiting | Dec 2031 |
| USA  NCT03256240^25^ | (550) | Kono-S vs. side-to-side functional end anastomosis | | Recruiting | Dec 2033 |
|  | *Patients with perianal CD…* |  | | | |
| USA  NCT01145365^26^ | …with one or more perianal fistulas (21) | Surgical drainage then certolizumab vs. certolizumab | | Completed | June 2015 |

**References**

1. Transanal Versus Transabdominal Minimally Invasive Proctectomy With Ileal Pouch-anal Anastomosis On Postoperative Outcomes in Ulcerative Colitis: a Randomized Controlled Trial. ClinicalTrials.gov. Identifier: NCT03536988. Updated 25 May 2018. Accessed 28 July 2025. Available from <https://clinicaltrials.gov/study/NCT03536988>
2. Is Diverting Loop Ileostomy Necessary in Completion Proctectomy With Ileal Pouch Anal Anastomosis: A Multicentre, Randomized Study of the GETAID Chirurgie Group. IDEAL Trial. ClinicalTrials.gov. Identifier: NCT03872271. Updated 8 February 2023. Accessed 28 July 2025. Available from <https://clinicaltrials.gov/study/NCT03872271>
3. Functional Outcomes of Transanal Ileal Pouch-Anal Anastomosis Compared to Laparoscopic or Open Ileal Pouch-Anal Anastomosis: a Multi-Center, Randomized, Parallel-Group, Non-Inferiority Trial. ClinicalTrials.gov. Identifier: NCT04722757. Updated 6 September 2023. Accessed 28 July 2025. Available from: <https://clinicaltrials.gov/study/NCT04722757>
4. ADVANCED-UC TRIAL: AppenDectomy Vs ANti TNF-a in Inducing Clinical and EnDoscopic Remission in Left-sided Ulcerative Colitis - A Randomized Clinical Trial. ClinicalTrials.gov. Identifier: NCT05931458. Updated 5 July 2023. Accessed 28 July 2025. Available from: <https://clinicaltrials.gov/study/NCT05931458>
5. Endoscopic Balloon Dilation as Compared to Surgical Management for the Treatment of Short Strictures in Fibrostenosing Crohns Disease: A Randomized Controlled Trial. ClinicalTrials.gov. Identifier: NCT03735355. Updated 8 November 2018. Accessed 28 July 2025. Available from: <https://clinicaltrials.gov/study/NCT03735355>
6. Prospective Randomized Comparative Study of the Treatment of Multisegmental Fibrostenosing Crohn's Disease. Surgical Resection Plus Endoscopic Stricturotomy Versus Surgical Resection Plus Strictureplasty. ClinicalTrials.gov. Identifier: NCT04865484. Updated 29 April 2021. Accessed 28 July 2025. Available from: <https://clinicaltrials.gov/study/NCT04865484>
7. SyMptomAtic Stricturing Small Bowel CRohn's Disease - Medical Treatment Versus Surgery, a Prospective, Multi-centre, Randomized, Non-inferiority Trial. ClinicalTrials.gov. Identifier: NCT05584228. Updated 18 October 2022. Accessed 28 July 2025. Available from: <https://clinicaltrials.gov/study/NCT05584228>
8. Surgical Intervention Versus Biologics Treatment for Symptomatic Stricturing Crohn's Disease (SIBTC): an Open-label, Single-center, Randomized Controlled Trial. ClinicalTrials.gov. Identifier: NCT05421455. Updated 13 July 2022. Accessed 28 July 2025. Available from: <https://clinicaltrials.gov/study/NCT05421455>
9. Laparoscopic ileocolic resection versus infliximab treatment of recurrent distal ileitis in Crohn's disease: a randomized multicenter trial - LIR!C. Overview of Medical Research in the Netherlands (OMON). Identifier: NL-OMON31330. Updated 13 May 2024. Accessed 28 July 2025. Available from: <https://trialsearch.who.int/Trial2.aspx?TrialID=NL-OMON31330>
10. Laparoscopic bowel resection combined with infliximab treatment versus infliximab for localized intestinal Crohn's disease: a randomized controlled, open-label trial. Chinese Clinical Trial Register. Identifier: ChiCTR2000029323. Updated 25 January 2020. Accessed 28 July 2025. Available from: <https://trialsearch.who.int/Trial2.aspx?TrialID=ChiCTR2000029323>
11. Early Surgery Versus Conservative Treatment in Patients With Ileocaecal Crohn's Disease - Prospective Randomized Study. ClinicalTrials.gov. Identifier: NCT02716454. Updated 28 April 2016. Accessed 28 July 2025. Available from: <https://clinicaltrials.gov/study/NCT02716454>
12. Mesenteric Sparing Versus High Ligation Ileocolic Resection for the Prevention of Recurrent Crohn's Disease. ClinicalTrials.gov. Identifier: NCT03172143. Updated 25 February 2019. Accessed 28 July 2025. Available from: <https://clinicaltrials.gov/study/NCT03172143>
13. Crohn's Extent of Resection Trial (CERT): A Randomised Controlled Trial Comparing Anastomotic Disease Recurrence Following 2cm Versus 10cm Resection Margins for Patients With Ileocolic Crohn's Disease. ClinicalTrials.gov. Identifier: NCT01876264. Updated 7 August 2020. Accessed 28 July 2025. Available from: <https://clinicaltrials.gov/study/NCT01876264>
14. Recurrence of Crohn's Disease After Ileocolic Resection Affected by Precisely Side-to-Side Anastomotic Type: a Randomized, Controlled Trial. Chinese Clinical Trial Register. Identifier: ChiCTR-INR-16008585. Updated 18 April 2017. Accessed 28 July 2025. Available from: <https://trialsearch.who.int/Trial2.aspx?TrialID=ChiCTR-INR-16008585>
15. Application of indocyanine green fluorescence angiography in preventing anastomotic recurrence after Crohn’s disease intestinal resection and anastomosis: a single-center, prospective, randomized controlled trial. Chinese Clinical Trial Register. Identifier: ChiCTR2000035737. Updated 1 October 2020. Accessed 28 July 2025. Available from: <https://trialsearch.who.int/Trial2.aspx?TrialID=ChiCTR2000035737>
16. Rct in croHn's Disease: Comparing mANual (End to End and Kono-s) Versus stapleD Side TO Side Ileocolic Anastomosis (HANDTOEND). ClinicalTrials.gov. Identifier: NCT05246917. Updated 31 May 2022. Accessed 28 July 2025. Available from: <https://clinicaltrials.gov/study/NCT05246917>
17. A randomised controlled trial to assess whether the amount of mesentery removed, or the type of bowel join used during surgery for Crohn's disease, can affect the chances of getting further disease. Current Controlled Trials. Identifier: ISRCTN16900055. Updated 13 November 2024. Accessed 28 July 2025. Available from: <https://www.isrctn.com/ISRCTNISRCTN16900055>
18. Optimising Surgical Anastomosis in Ileocolic Resection for Crohn's Disease to Reduce Recurrent Disease: A Randomised Controlled Trial Comparing Hand-sewn (END-TO-END or Kono-S) to Stapled Anastomosis (END-to-END Study). ClinicalTrials.gov. Identifier: NCT05578235. Updated 18 December 2023. Accessed 28 July 2025. Available from: <https://clinicaltrials.gov/study/NCT05578235>
19. MeSenteric SpAring Versus High Ligation Ileocolic Resection for the Prevention of REcurrent Crohn's DiseaSe (SPARES). ClinicalTrials.gov. Identifier: NCT04578392. Updated 5 April 2022. Accessed 28 July 2025. Available from: <https://clinicaltrials.gov/study/NCT04578392>
20. KONO-S Anastomosis Compared to Conventional Ileocolonic Anastomosis to Reduce Recurrence in Crohn's Disease: a Superiority Phase III Prospective, Randomized, Multicenter, Double-blind Trial. ClinicalTrials.gov. Identifier: NCT05974358. Updated 13 February 2025. Accessed 28 July 2025. Available from: <https://clinicaltrials.gov/study/NCT05974358>
21. Extended Mesenteric Resection in Ileocecal Crohn's Disease to Prevent Recurrent Disease - A Randomized Controlled Trial. ClinicalTrials.gov. Identifier: NCT06324838. Updated 4 April 2024. Accessed 28 July 2025. Available from: <https://clinicaltrials.gov/study/NCT06324838>
22. Stapled Antimesenteric Functional End-to-end Anastomosis (Kono-S) Versus Stapled Antimesenteric Isoperistaltic Side-to-side Anastomosis for the Postoperative Recurrence of Crohn's Disease: a Randomized Control Trial Study. ClinicalTrials.gov. Identifier: NCT05658081. Updated 20 December 2022. Accessed 28 July 2025. Available from: <https://clinicaltrials.gov/study/NCT05658081>
23. A multicentre randomised controlled trial of the effect of the modified Kono S anastomosis versus the stapled side to side anastomosis on disease recurrence after ileocolic resection for Crohn’s disease. Australian New Zealand Clinical Trials Registry. Identifier: U1111-1276-0767. Updated 6 April 2025. Accessed 28 July 22025. Available from: <https://anzctr.org.au/Trial/Registration/TrialReview.aspx?ACTRN=12622000809730>
24. Mesentery Guided Bowel Resection Margin Versus Traditional Margin in Reducing Early Endoscopic Recurrence Rate After Ileocolic Resection in Patients With Crohn 's Disease: a Prospective, Multicenter, Randomized Controlled Clinical Trial. ClinicalTrials.gov. Identifier: NCT06241170. Updated 15 July 2025. Accessed 28 July 2025. Available from: <https://clinicaltrials.gov/study/NCT06241170>
25. Prospective Randomized Study of the Kono-S Anastomosis Versus the Side-to-side Functional End Anastomosis in the Prevention of Post-operative Recurrence of Crohn's Disease. ClinicalTrials.gov. Identifier: NCT03256240. Updated 27 January 2025. Accessed 28 July 2025. Available from: <https://clinicaltrials.gov/study/NCT03256240>
26. A Prospective Multicenter Trial Evaluating the Benefit of INitial Surgically Established Drainage Prior to Medical Therapy for the Treatment for Crohn's Perianal Fistulas. ClinicalTrials.gov. Identifier: NCT01145365. Updated 4 April 2017. Accessed 28 July 2025. Available from: <https://clinicaltrials.gov/study/NCT01145365>
